# Water Insecurity in the Jordan Valley: Community Perspectives on Its Impacts on Maternal and Child Health †

**DOI:** 10.3390/ijerph22020187

**Published:** 2025-01-28

**Authors:** Antonia Walther, Amira Shaheen, Hamza Zubeidat, Ghassan Shakhshir, Shakoor Hajat

**Affiliations:** 1 Centre on Climate Change and Planetary Health, London School of Hygiene and Tropical Medicine, London WC1E 7HT, UK; amira.shaheen@najah.edu; 2 Dr. von Hauner Children’s Hospital, Ludwig Maximilian University, 80337 Munich, Germany; 3 Public Health Department, Faculty of Medicine and Health Sciences, An-Najah National University, P.O. Box 7 Nablus, Palestine; 4 Ma’an Development Centre, P.O. Box 51352 Ramallah, Palestine; 5 Development Cooperation Unit, Norwegian Representative Office to PA, P.O. Box 25161 Jerusalem, Palestine; ghassan.shakhshir@mfa.no

**Keywords:** WASH, water insecurity, child health

## Abstract

The Jordan Valley in the West Bank in Palestine provides a unique social, environmental, and geopolitical context in regard to the global challenge of water insecurity, where its impacts on child and maternal health are only partly understood. Existing research has been largely limited to investigations of water quantity/quality and direct health outcomes, such as infectious disease. This qualitative study aimed to provide a holistic perspective of the challenge of water insecurity and child health, by investigating household water insecurity in Palestine. Focus group discussions explored the lived experiences of women from marginalized communities. These were then thematically analyzed, in reference to social theory. The study identified context-specific aspects of water insecurity, shaped by the background in Palestine involving the occupation and ongoing violent conflict in the area. These challenges disproportionately affect women, who are primarily responsible for water management within their communities, leading to embodied experiences, heightened negative emotions, and increased conflict, both within households and the broader community. Consequently, these stressors heavily impact children: limited caretaking time, due to economic pressures, children’s involvement in water-related tasks, and the disruption of social cohesion at both the community and household level, ultimately affect their physical and mental health and their ability to learn and play. Our findings could guide research and policy efforts in developing context-sensitive tools, such as a child water stress index for Palestine.

## 1. Introduction

The complex interplay of geopolitical circumstances, environmental stressors, and cultural factors provides a unique context within which to consider the implications of water insecurity for children in the Jordan Valley. Water security is central to human security and sustainable development and is closely linked to public health concerns [1,2,3]. The states around the Jordan Basin (Jordan, Palestine, and Israel) are especially vulnerable to water insecurity, due to local environmental conditions and geopolitical instability [4]. In Palestine, naturally high levels of water stress are aggravated by its occupation, population growth, and climate change, with subsequent water demand expected to increase by up to 40% in the coming decades [5,6]. Complex water-sharing agreements also exacerbate water insecurity in this region [4]. The utilization of transboundary water resources, such as the Mountain Aquifer and the Jordan River Basin, is regulated by agreements established under the Oslo II Accords [4]. However, the region’s political instability hinders the effective implementation of these agreements and the imbalance of political power results in the unequal distribution of water resources between Israel and Palestine [4,7]. For instance, Palestinian applications to the Joint Water Committee, which oversees the allocation of water from the Mountain Aquifer, have faced repeated delays and have a significantly lower approval rate compared to Israeli applications [4,7].

**Figure 1:**
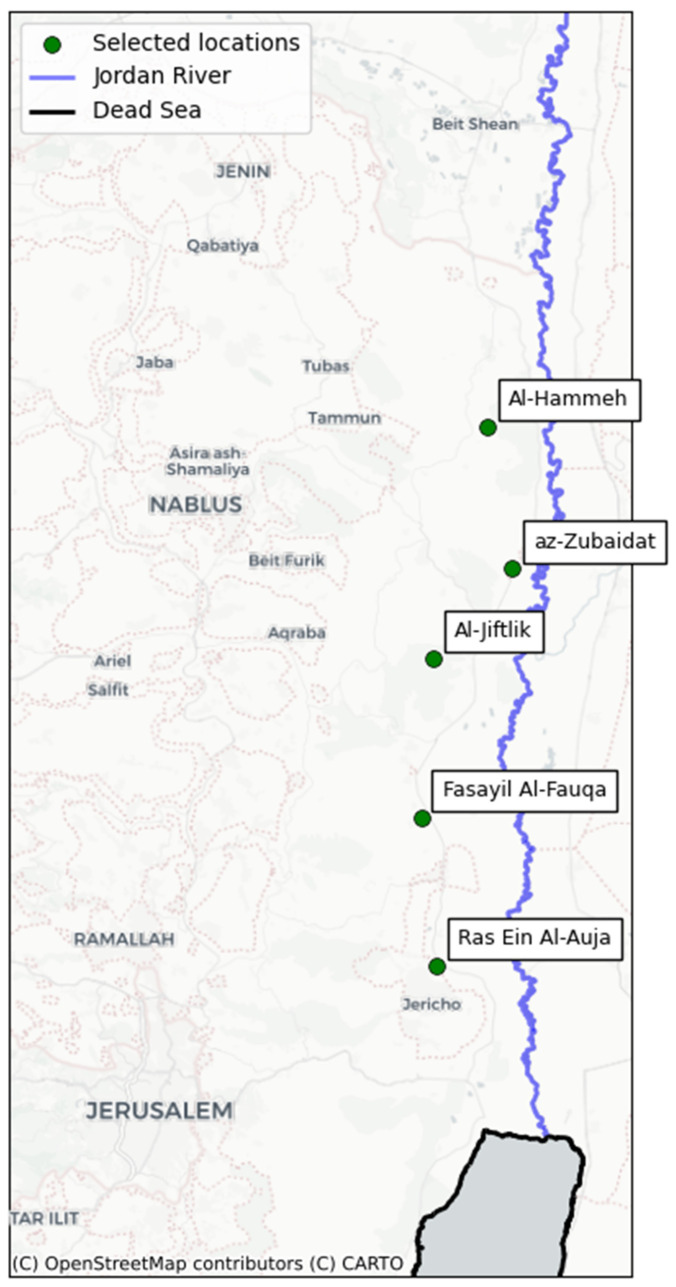
Map of the Jordan Valley, with marked study sites, including the pilot FGD. Map data copyrighted OpenStreetMap contributors and available from [11].

Consequently, resources like the Jordan River, despite being in the West Bank (see Figure 1), provide only limited benefits to the local population. This is aggravated by frequent displacement, the ongoing occupation, and population growth [3,4,5,8,9,10].

Local conflict also complicates water transport and its costs [3,12]. Despite high levels of poverty, especially in the Jordan Valley, Palestinians spend up to one sixth of their household budget on buying water from official or private providers [12]. High water expenses make it especially hard for herder communities, like Bedouins, to maintain their traditional lifestyle [12,13]. The 15,000 Bedouins living in the Jordan Valley today are displaced indigenous tribes, who have been expelled from their original lands in the Negev desert [14]. Having settled in areas suitable for their semi-nomadic lifestyle, herding and agriculture are central to their livelihood and cultural identity [14]. However, they mostly live in unrecognized villages that lack water, sanitation, and hygiene (WASH) infrastructure, schools, and healthcare [15,16].

In addition to being a central source of conflict in the region, water insecurity leads to severe health consequences. Globally, it is estimated that improving WASH could reduce child mortality from infectious diseases by 45% [17,18]. In rural Palestine, at least 16% of drinking water derives from unimproved water sources and 80% of the West Bank is not connected to the sewage system, leading to a high risk of waterborne diseases [3,19]. Insufficient WASH practices and a high prevalence of parasitic diseases in the rural West Bank indicate that challenges faced by marginalized communities are under-reported [20]. In these populations, women and children disproportionally suffer from water insecurity, but little is understood about the impacts on children beyond their direct physical health [8,21,22]. In their role as caregivers, women mediate child health, but also experience a physical and psychological burden themselves due to the lack of WASH facilities [21,23,24,25]. The success of WASH is connected to technological and psychosocial conditions, in the context of culture, social hierarchies, and interpersonal relationships [26,27]. Social structures and resilience within the community are weakened by a lack of resources and children are impacted by the socio-political effects of water insecurity [3,8,23,28,29]. Community resilience is closely tied to “social capital”, which refers to the ability to effectively engage with individuals both within and outside the community, as well as with institutions, such as government bodies [30]. When natural resources, such as water, are scarce and governments fail to meet the population’s basic needs, the risk of crime, violence, and rebellion increase, further deepening ethnic and social divides [28,30].

Very few studies have investigated the interplay between water insecurity and child health in Palestine [31,32]. Data from the West Bank indicate that 94% of children have access to clean drinking water, however the numbers on the health burdens faced by these children suggest otherwise [31]. High rates of stunting (16%), anemia (49%), and parasitic disease (16%), as well as eye infections, respiratory tract infections, and diarrhea have been reported, especially among Bedouin communities in the Jordan Valley [13,20,33,34]. A recent assessment of individual water insecurity in Palestine found that 50% of the poorest women experience water insecurity, with over half of the population expressing concerns about water access [35]. However, these data encompass Palestine in general and it remains unclear whether marginalized populations, such as those in the Jordan Valley, were included. Moreover, the experience of children remains unknown.

This lack of information indicates that established indices assessing water insecurity are insufficient in regard to this population. The majority of existing studies use quantitative methods to measure the impacts of physical features, whilst less attention is paid to household dynamics [36]. Indices have recently added access, cost, reliability, lifestyle/culture, and equity to commonly used aspects of quantity and quality [37,38]. These factors play out on two levels: the macro perspective includes the quantity and quality of water, livelihoods, and ecosystems; whilst the micro perspective refers to the household/individual [23]. Here, we concentrate on the micro perspective; the conceptualization of household water insecurity requires an in-depth assessment based on lived experiences, requiring the use of qualitative methods [23,39,40]. We primarily focus on the behavioral responses of women to water insecurity and their impact on children within the community. The relationship between water insecurity and child health is a dynamic and complex system that continuously adapts to its context and where consequences are a result of interactions [41]. Here, health is understood as a holistic concept of well-being and, so, this study explores factors beyond physical health, such as psychosocial effects. By focusing on a local perspective from within the community, we aim to contextualize water insecurity, define local aspects, and understand its implications for maternal and child health.

## 2. Materials and Methods

An exploratory qualitative approach involving focus group discussions (FGDs) was chosen, focusing specifically on interpersonal dynamics and the community’s values. Focus groups allow participants to respond in their own words and to choose discussion topics themselves [42]. Participants may challenge and stimulate the responses of others, thus facilitating an honest discussion and creating room for spontaneous responses. Moreover, considering the logistical challenges due to the ongoing local conflict in the region and the time restraints on the participants, focus groups were deemed an effective way to gain insight into a range of opinions and perceptions.

Fieldwork and data generation were carried out over the course of four weeks in July 2023, by two of the authors, AW, who is female, and HZ, from the local organization “Ma’an”, who is male. “Ma’an” have long-standing experience in the Jordan Valley, working on environmental projects, advocacy, and female empowerment. HZ is a native Arabic speaker and is local to the study site, with long-standing experience of collecting field data. The Consolidated Criteria for Reporting Qualitative Studies (COREQ) were followed (see Supplementary Materials) [43].

### 2.1. Conceptual Framework

The conceptual framework for this study was guided by three concepts of social theory. Political ecology of water conceptualizes the human and political dimensions of water insecurity regarding: (i) the levels of access, (ii) the patterns of use and control; and (iii) that the cultural and economic value of water contributes to a comprehensive understanding of the situation [44]. These dimensions are shaped by the interests and roles of the individuals affected, ultimately determining the consequences of water insecurity. According to feminist political ecology, interests in natural resources are gender-related, based on “distinctive roles, responsibilities and knowledge” ([45], p.522). Gender plays a crucial role, which necessitates an exploration of everyday water-related practices linked with oppression, discrimination, and exploitation [46].

Finally, the effects on child health can be placed within the concept of early childhood development (ECD), considering the following levels [47]: (i) personal refers to the child and its characteristics, (ii) process includes daily interactions with caregivers, such as through learning, reading books, singing, and playing; (iii) time involves events and changes during the child’s lifetime [23,47]; and finally, (iv) context is separated into different levels, such as the household, the community’s influence, such as the parent’s work, social services, and the social, economic, and demographic background [23,47,48].

### 2.2. Study Sites and Participants

Since households in the Jordan Valley experience wide variation in regard to their experience of water insecurity, four FGDs in different locations were conducted (see Figure 1). All the study sites are isolated from major cities, have limited access to electricity and a clean water supply, and lack public transportation, health, and education services, as well as waste disposal. The population can utilize one primary healthcare centre in Al-Jiftlek and hospitals in Nablus, Tubas, and Jericho, which are a 30–40 km drive from the studied communities.

In total, 23 participants (excluding those involved in the pilot FGD) took part. The FGDs consisted of 3 to 11 women, as a recommended group size [49] The inclusion criteria were being female and over 18 years of age, having lived in the selected community for at least one year, and being in a caregiving role (mother, aunt, grandmother). Communities were purposively selected based on the advice of Ma’an, capturing diversity in terms of local ecology and water insecurity. Community leaders were contacted by phone and introduced to the study. If they agreed, in a next step, following convenience sampling, women were approached face-to-face and were invited to participate.

The subjects came from four different communities; the details on the composition of the FGDs can be found in Appendix A. All the participants were from farming households and every woman who agreed to participate was invited. This offered everyone the possibility to have their views and perceptions included in the analysis. Moreover, as advised by local researchers, the arbitrary exclusion of a few community members could be misunderstood and lead to a lower level of acceptance of the study. In total, two women did not agree to participate, due to time constraints and caretaker responsibilities.

### 2.3. Data Collection

To generate a naturalistic environment, the participants themselves decided whether to gather for the discussions at home or in a public space (council hall) [49] Following the pilot FGD, open-ended questions asking the participants to describe their experiences of water issues and the health status of women and children were adapted. To ensure acceptability and transparency, an introductory session lasting 30–60 min was conducted with both male and female community members. During the FGDs, however, only female participants were present and the male community members were excluded. This approach supported the need for open discussions on private and potentially sensitive topics. The FGDs were conducted in Arabic and audio recorded over 60–90 min. After each discussion, the researchers (AW and HZ) visited the location and its water source(s) to gain a deeper understanding of the context. Fieldnotes on the participants’ reactions, the environmental conditions, and personal reflections were continuously taken. The audio recordings were then translated and transcribed by a Palestinian professional translator. To ensure correct translation, the transcript was additionally validated by two of the authors (HZ and AS), both native Arabic speakers.

### 2.4. Data Analysis

Reflexive thematic analysis was performed, according to the work of Braun and Clarke and following a three-level coding process [50,51]. First, open coding was conducted on each FGD individually, to inductively generate ideas from the data [51]. Two researchers reviewed the data, developing initial ideas, which were then grouped into preliminary codes. Subsequent focused coding and theme development was conducted by one researcher, since the project was designed for a single lead investigator (AW). During this phase, significant codes were identified, disconnected codes were refined, and overarching ideas were characterized. Focused codes were operationally defined considering the fieldnotes and accounted for contradictory findings as negative cases [50]. Finally, the focused codes were grouped into themes, aligned with the study’s social theory framework. This iterative process ensured each code belonged to a single theme and relationships between themes were established. For example, the quote, “*You know, asking one time is fine, the second is okay, but then the third time you will feel ashamed and you will stop asking for water and have to get the water from the spring*”, was initially coded as “asking for water”. This evolved into the focused code, “being a burden”, and, ultimately, the theme “shame”. This theme highlights distinct roles in terms of water access, power dynamics, and economic disparities, reflecting elements of the conceptual framework.

No statistical analysis was performed. Due to the nature of the FGDs and the local dialect, the transcript included many unfinished sentences and comments. In order to adapt the analysis to the data, no specialized software was applied. The transcripts, codes, and themes were organized in Excel.

## 3. Results

### 3.1. Context Specific Specific Aspects of Water Insecurity in the Jordan Valley

**Figure 2:**
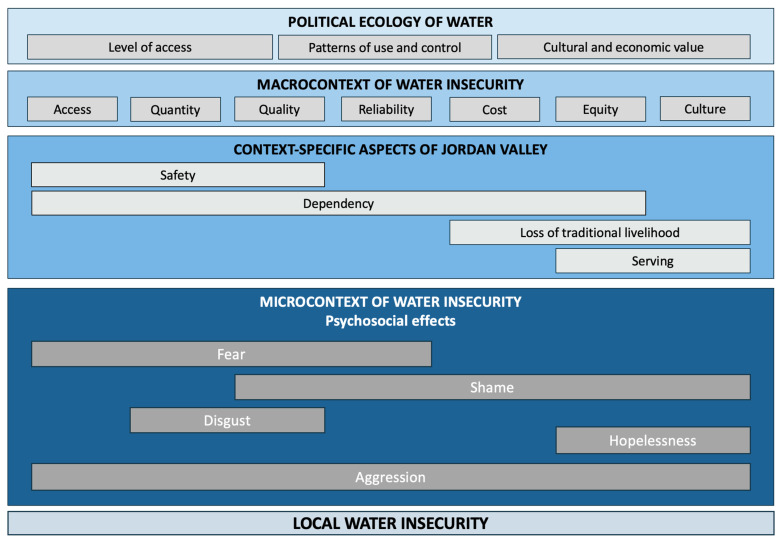
Context-specific aspects of water insecurity and the associated macro- and micro-level variables. Emotions perceived within the communities are linked to these variables. For instance, in the Jordan Valley, “safety” influences factors such as water quantity, quality and access, with participants associating “fear” with this variable.

Context-specific aspects shape water insecurity in the Jordan Valley in a unique way and are summarized in Figure 2. The defined themes can be allocated to the central ideas in the political ecology of water and relate to the established categories of quality, quantity, access, cost, culture, reliability, and equity.

Levels of access to water in the Jordan Valley are mainly related to safety, as mentioned in all the FGDs: “*(Israeli) settlers take over the water spring—they destroy the place; they don’t let anyone anywhere near the water*” (FGD1). Safety includes threats on the way to or at the water source, the confiscation of tractors when transporting water, and safety in terms of working with outside water tanks at night. Safety’s impact on the quantity, reliability, access, and cost of water is related to the geopolitical macro context involving the occupation in the Jordan Valley.

Water sharing was widely practiced in the communities, generating a feeling of dependency. The practice of “asking for” water (from husbands or other households) enforces social and economic differences within the community. Water is, thus, related to economic power and good health is viewed as a privilege: “*Some people don’t have tractors to get the water, they pay 100 shekels to go to Al-Auja and get water. Because of that I get water from the neighbors*” (FGD1).

In addition to direct water expenditure, costs for the maintenance of water infrastructure or the treatment of waterborne diseases were mentioned. This links water insecurity with economic stability, which affects equity and reliability.

Water insecurity further impacts traditional livelihoods when farmers are forced to sell their livestock: “*Most of the families here sell their sheep because there’s not enough water*” (FGD1). Some reported stopping farming or planting specific products in contradiction with traditional farming: “*When we were kids there were many orchards, we (…) used to plant all kinds of vegetables, banana orchards, there was a tap of water in every house (… but) nowadays, no more orchids, no more variety in vegetables, there are fewer farmers because the water is less, most are cultivating dates now because they don’t need a lot of water and it pays more*” (FGD4). The resulting lower level of connection to the land and negative personal reputations were a substantial worry: “*It has turned into a (bad) reputation of Al-Jiftlik to mix water (used for cultivation) with sewage water*” (FGD4). This leads to lower value products and the land was linked by participants to cultural values.

Water also shapes cultural identity through its role in serving practices, such as religious rituals that require hygiene: “*Water is important for the human body (…) and bathing is sacred to me*” (FGD3). Acts of hospitality, such as serving guests, were perceived as a basic need, which in some cases were more important than personal needs. Participants reported that they would refrain from asking their neighbors for water for themselves, but would do so if they had guests: “*If you have a guest it (lack of water) holds you down, with everything*” (FGD2).

Overall, water insecurity in the Jordan Valley is impacted by safety issues, intra-community dependencies, and alienation from the traditional lifestyle. Water insecurity, thus, reshapes social hierarchies in the studied communities.

### 3.2. Psychosocial Implications for Women

#### 3.2.1. Roles and Responsibilities

Water insecurity is reflected in women’s roles and responsibilities and, consequently, in their emotions. The FGDs confirmed that women are mainly responsible for the management of water within the household and the community: “*The men are responsible for getting the water tanks and then it’s all on us women, and most importantly we have to save*” (FGD1).

Sending male community members to bring water from either a distant or unsafe source generates fear among the female members: “*When our men go to the water, the women wait anxiously. We wait in fear*” (FGD1). Furthermore, constant surveillance by Israeli drones when leaving home to access WASH facilities renders the “outside” environment unsafe. The lack of privacy and the pervasive surveillance in these spaces, which are also the locations of water sources and WASH facilities, enhance the feeling of fear. This fear undermines women’s confidence to work in such environments, despite these areas being vital for accessing essential resources.

As water managers, women take on responsibilities that are deeply influenced by societal expectations. The failure to fulfill these expectations often results in feelings of **shame**. Two aspects contribute to this feeling. The first aspect relates to being blamed for the lack of resources: “*He would tell me why haven’t you saved it, we would’ve had more water if you don’t waste it*” (FGD1).

The second aspect relates to shame due to their position in society, illustrated by the need to ask for shared water, or feeling unclean: “*Asking one time for water is fine, the second is okay, but the third time you will feel ashamed...*” (FGD1).

#### 3.2.2. Embodied Experiences

In addition to mental stressors, embodied experiences enhance negative emotions. Lacking reliable information on water quality and the risk of disease, women rely on instinctive associations with dirty or smelly water. The feeling of disgust prevailed. Some women reported that they prefer to stay thirsty to avoid the compelling sensation of drinking unclean water: “*By seeing the cloudy water, filled with dirt, you mentally become sick, you are compelled do drink it (…) Adults would endure but children wouldn’t*” (FGD1). Consequently, chemicals, although perceived as unhealthy, are needed to sanitize the water. This complicates the inner struggle involving environmental needs that are in contradiction with personal needs.

In all the FGDs, the physical labor related to water and its effects on women’s bodies were discussed. Interestingly, the struggle in regard to accessing, demanding, and using water was summarized by one participant as the “*long way to water*” (FGD1). This refers not primarily to distance, but describes the obstacles between her (body) and the resource. Moreover, the workload involved and the time spent dealing with water lead to the experience of pain and exhaustion. Sleep deprivation due to chronic pain and worry were frequently mentioned. As a result, water management creates hopelessness. Running out of water and, therefore, putting family members at risk is a constant fear, as well as asking for water from their husbands or neighbors, since this means endangering them or generating additional costs. The ongoing and recurring nature of water insecurity hinders the possibility of achieving a lasting solution. Women reported a loss of hope, particularly in the context of political oppression and occupation.

In addition to the overall negative and paralyzing emotions hindering the individual from tackling challenges, all the FGDs contained the notion of aggression: ***“****We are ready to explode in anyone’s face!”* (FGD3).

In summary, water insecurity affects women’s mental health, their bodies, and their identities. In Figure 2, the relationship between the themes is displayed. For instance, dependency restricts access to water by evoking negative emotions, such as shame and fear. Those afraid to approach water sources directly because of safety may rely on water from their neighbors, but may feel too ashamed to ask. This dynamic operates outside traditional power structures and socio-economic privilege, and cultural hierarchies are reshaped by the power linked to water.

### 3.3. Implications for Child Health

**Figure 3:**
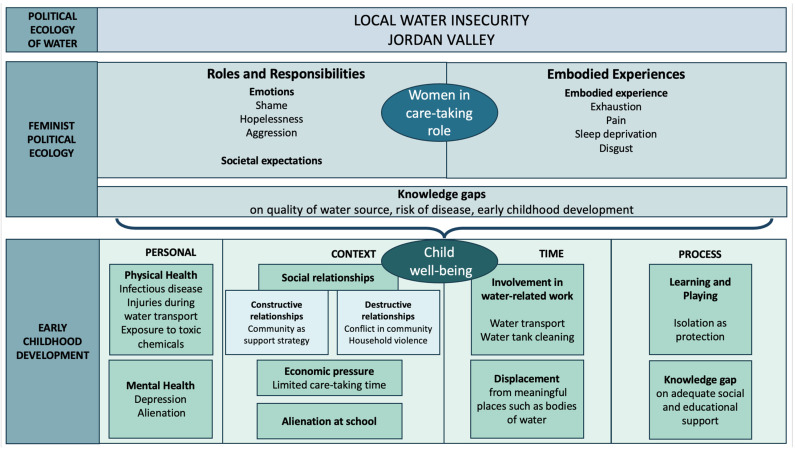
Implications of local water insecurity in the Jordan Valley. On the left, social theories that informed the analysis are outlined. The impacts of water insecurity on women, along with context-specific factors, influence child health, considering the four dimensions of early childhood development.

Household and community dynamics impact the caretaker’s ability to sustain adequate processes, such as child care, nurturing, and health. In this analysis, these effects are conceptualized in regard to the theory of early childhood development (see Figure 3).

#### 3.3.1. Context

The context is shaped by social relationships and their impact on the child’s environment. The emotional and embodied experiences of women induced two contradictory processes in terms of social relationships, namely a constructive process versus a destructive process, which plays out in a conflict-provoking way. In the former, conversing over water problems was described as helpful and relationships among women were perceived as a source of support. However, where water insecurity became overwhelming, relationships and communication represented rather a “valve” for internal pressure and frustration, causing conflict in the community among the women and at home: *“The other day on Eid day, my aunt and my sister were arguing a lot because of the water problem because they fought on who’s turn it is”* (FGD3).

Conflicts of interest in regard to water usage and levels of dependency, as described above, introduce a power dynamic into relationships between women from different households, but also between wife and husband: *“We always fight over water, it affects our relationship, all of our fights are because of water”* (FGD1).

Finally, conflict arises between parents and children: “*Because of the constant stress, we would attack the weaker*” (FGD3). Mothers revealed that their aggression is directed against “the weaker” members of the family, as in men towards women and women towards children. This notion of household violence in relation to water insecurity poses a serious threat to child well-being. Furthermore, various accounts involving the explicit mentioning of children’s mental distress at school due to water insecurity were collected. For example, alienation at school due to social disparities was reported: *“(Those) students are alienated in the class, others can’t tolerate their smell and (…) this generates negative feelings, they say bad words and fight. They might respond with violence”* (FGD4).

Economically and demographically, children became victims of their families’ financial insecurity, driven by the high costs associated with water. Since women need to contribute to the family income, they work in Israeli settlements, which limits the time for caretaking: “*I don’t have time to help them study, or feed them, nothing*” (FGD3).

#### 3.3.2. Personal

At the personal level, reports of diseases, such as fever, diarrhea, and eye, respiratory, and skin infections, were consistent across all the FGDs. In all the discussions, a recent presentation to health facilities due to water-borne disease was mentioned. Crowded living conditions and an unclean household, due to a lack of sewage and waste disposal systems, were perceived as the main health-related threats. In some communities, boys bear the responsibility of undertaking the hazardous journey of transporting water. One participant expressed her worry, saying, *“Oh, to think about my son driving the tractor one day…! I fear the day he may not come back; he’s 13 years old”* (FGD1).

Additionally, the constant stress related to thirst, feeling dirty, and the wish to cool off in the hot environment was a major concern. Various accounts of mental distress were collected. Children feel nervous, depressed, unclean, and worthless, indicating that water insecurity affects children’s mental health and self-esteem: “*Of course, it affects their upbringing, they become depressed*” (FGD3).

#### 3.3.3. Time

Children’s daily routines are disrupted by their involvement in water-related tasks, which are often perceived as unsafe. They are involved in water transportation, as well as in cleaning water tanks using chemicals.

“*They get tired from carrying the water over and over again*” (FGD1). A further disruption to children’s lives relates to their displacement from meaningful places, such as their own water source, for instance, in the case of the confiscation of water sources.

“*The most problematic days are Fridays and Saturdays (when we used to play at the water) because the settlers take over the water spring*” (FGD1).

#### 3.3.4. Process

Women acknowledged that spending time outside and among friends is important for children. Yet, they were hesitant to support this, fearing exposure to dust, heat, and water-borne disease. Perceiving the outside as unsafe and correlated with fear, participants considered it beneficial to keep children inside the house: “*In winter I hide them inside, and the same in summer*” (FGD2).

Thus, children were isolated, preventing them from social interaction, learning, and playing. Furthermore, despite being aware of the links between WASH and child health, the participants were worried about their own lack of knowledge on how to best protect and support their children:

*“The way they start to talk with me (disrespectful), they are not good in school, one can’t pronounce a lot of letters, one is slow in learning, I don’t know why, I took him to the hospital, they couldn’t help me…”* (FGD3).

In summary, the findings show how water insecurity affects various aspects of early childhood development and that it presents a significant challenge to child well-being, extending beyond physical health.

## 4. Discussion

This study highlighted the context-specific aspects of water insecurity in the Jordan Valley, such as safety, dependency, loss of traditional livelihood, and service shaped by the occupation and ongoing conflict. Women are disproportionally impacted by these challenges as a result of embodied experiences and negative emotions, such as fear, shame, disgust, hopelessness, and aggression. The resulting conflict in the community and at home disrupt social cohesion and reshape social hierarchies. This extends to impacts on child well-being, beyond physical health, affecting all aspects of early childhood development. Water insecurity contributes to feelings of depression and exposure to violence, disrupting daily routines through involvement in water-related tasks, and hindering children’s ability to learn and play.

Context-sensitive variables capture how water insecurity is mitigated, according to contextual and individual possibilities to adapt. Their relevance in assessing water insecurity has been shown before. For example, the analysis of water insecurity in Egypt, considering context-sensitive variables, yielded estimates 27 times higher than those reported by the Joint Monitoring Programme, which did not account for such factors [39].

Safety is part of the Household Water Insecurity Experiences (HWISE) Scale and has been reported in other contexts as well [52]. Yet, this aspect mainly refers to the physical threat of low quality water or sexual assaults when transporting water or intimate partner violence due to conflicts over water [52]. To the best of our knowledge, the association with political and military threats has not been defined in the context of water insecurity assessments. Since it was a central theme in our study, our results can inform the development of a child water stress index for Palestine [23], possibly extending the HWISE [39].

These external factors have an impact on women, who have been shown to be more likely to be affected by emotional stress due to household water insecurity [22,53,54,55]. Sultana et al. conceptualized the physical, mental, and social consequences of water insecurity as embodied subjectivities to describe how an individual woman is inscribed with daily lived experiences, which then further affect her environment [46,56]. In the Jordan Valley, embodied consequences, such as constant pain and sleep deprivation, are comparable to other contexts [57,58,59,60]. Regarding psychosocial effects, the mental health burden of water management, interpersonal conflict, shame, and fear of harm, as observed in this study, have been related to water insecurity elsewhere [57,58,61]. However, gender-related roles in the Jordan Valley vary from the global picture, based on data from Sub-Saharan Africa and South Asia [37,38,57,58,62,63]. Water transport for example is considered a gendered aspect [57]. Contrary to other contexts in terms of water insecurity, in the Jordan Valley, the water source is close in proximity, but challenges are present in terms of obstacles to access or usage.

As reflected in the negative emotions elicited by water insecurity, we agree with Sultana et al. that women are “suffering for and from water” ([59], p.439). The emphasis on suffering relates to women’s agency to make informed choices depending on access to information, as well as the equal opportunity to act accordingly [64]. Our results show that women are eager to acquire knowledge on water quality, management, healthcare access, and child support, but do not know where or how to access this information. It has been shown that women feel empowered to make informed decisions at home/in the community after being provided with WASH-specific knowledge [65]. Encouraging community members to engage in decision-making leads to empowerment and better outcomes [65,66,67]. Dependency and water sharing has been observed in other contexts as a “shared struggle” regarding reciprocal water borrowing [57]. The introduction of participatory processes, such as jointly prepared WASH budgets, could address the issue of social hierarchies constructed as a result of water insecurity, drawing on already practiced water (and burden) “sharing” [64].

Finally, research on water insecurity and child health largely focuses on effects such as infectious diseases (diarrhea, respiratory tract infections, skin and eye disease), outcomes that have been confirmed by our participants’ accounts. Infectious diseases and safety-related injuries during water transport and contact with hazardous chemicals include physical threats to child health, as reported in the FGDs. This finding is in agreement with other studies [68]. However, there are many adverse consequences beyond infectious diseases. This study, guided by the theory on early childhood development, focused on the impact on children’s mental health, as well as their ability to learn and play [47].

In contrast to the vast literature on water insecurity and mental health among women, there is little research focusing on the psychological consequences for children. In this study, participant accounts contained explicit mention of mental ill-health and depression among children. Water insecurity that causes maternal depression may impact children’s mental health even further. Depressive symptoms in mothers were found to be the best predictor of their child’s morbidity [54,57,68,69]. There have also been indications that the shame of attending school unwashed, as a “social failure” or, as described here, “alienation at school”, is linked to mental ill-health [53,68]. Further, water insecurity when perceived as material deprivation, as in our study, is associated with depression in children [68,70].

Carrying the emotional and embodied burden of water insecurity, women convey the continuous pressure of water insecurity in the context of their children’s development. The presence of conflict within the household is aggravated by the lack of practical solutions and knowledge on fostering child well-being. Interpersonal conflict does not only reflect women’s reactions to water insecurity, but may lead to “household chaos” when caregivers experience distress ([23], p.385). In this study, aggression was mentioned regularly, raising concerns over household violence against children and women [68,71]. The intergenerational transmission of violence in association with water insecurity has been described in other contexts, mostly in Sub-Saharan Africa [52]. This may refer to pressures on children involved in water-related work, abuse or physical injuries while transporting water, or psychosocial stress within the household [52].

As opposed to our findings, in other studies, caretakers’ time-constraints were said to be a reason for income loss, due to the amount of time spent managing water [72]. In the Jordan Valley, economic pressures force women to work, which leads to less time for caretaking.

The domain of time in early childhood development refers to events and changes during the child’s lifetime. Water insecurity resulting in the disruption of daily routines and violence endanger children’s well-being [22,23]. Children are involved in water-related work, which involves associated health risks, such as the exposure to chemicals while cleaning water tanks, as reported in our study [60,68]. Furthermore, restricted access to, or displacement from, meaningful places, such as lakes and rivers, which is part of everyday life in the occupied West Bank, is also associated with mental ill-health in children [68,73]. The changes in cultural identity due to alienation from traditional livelihoods and cultural norms and values also shape the course of a child’s lifetime. It has been stated that psychosocial distress is rooted in the disparity between cultural and social norms in terms of water use and the barriers to access [58].

Finally, process describes the ability and necessity to learn and play in early childhood development. Due to economic restrictions, dependency, and knowledge gaps, children in water insecure communities are not adequately supported to thrive. Children’s desire to play outside (with water), contradicting the mothers’ fear of the “outside”, due to dangers from unsafe water, dirt, and dust, has been shown in one other study [74]. Yet keeping children “inside” undermines their opportunities and right to play, which is known to be fundamental for early childhood development [68,75].

The apparent multi-layered impacts of water insecurity on community life regarding mental health, social cohesion, household economy, and people’s sense of identity and political inclusion, observed in our study, are in accordance with other studies [58,68,71]. Our findings indicate that established indices of water insecurity are not fit for this unique context. The results further confirm the relevance of water insecurity not only for women’s, but also children’s, mental health and development.

## 5. Strengths and Limitations

Qualitative research is a subjective and reflexive process, prompting concerns regarding validity, rigor, risk of bias, and generalizability. This study is based on a small sample size, primarily due to the small household sizes (10–20 households) within the targeted communities, limiting the number of potential participants. Additionally, not everyone was available to join the discussions, due to daily responsibilities. However, in qualitative research, sample size is not the decisive element in the design of a study [76]. In this case, the small sample size enables a deeper exploration of the datas’ complexity, capturing nuanced and context-specific insights. The novelty and value of this study is not due to it being a representative sample of communities in the Jordan Valley, but rather to the exploration of new themes and concerns of a marginalized population.

The main challenges in regard to participant recruitment were logistical and safety related. The communities are located in remote areas that are often affected by violent conflict, making access difficult. Several planned meetings had to be canceled because of ongoing military operations, either along the route to the communities or within them. In some cases, meetings were also canceled due to sudden expulsion orders. As a result of these challenges, it was not possible to obtain participant feedback on the analysis.

A potential power imbalance between the researcher and the participants might have led to social desirability bias. However, the chance of obtaining sincere accounts was increased by collaborating with a local RA from Ma’an. To improve the validity of the analysis, the translations were verified by two listeners. During the analysis, the themes were checked for consistency with ideas noted during earlier reflections. To our knowledge, there has been no prior scientific study on water insecurity and health based on the perspectives of the affected population in the Jordan Valley and this research offers new insights beyond established evidence.

## 6. Conclusions

Water insecurity in the Jordan Valley is shaped by the political and military occupation and ongoing violent conflict in the area. Women are disproportionately experiencing negative emotions, physical violence, and increased conflict, both within households and the broader community. These stressors impact children’s development. Few studies globally have investigated the associations with water insecurity; however, there is growing evidence of its impact on children’s mental health and development. Limited caretaking time due to economic pressures, children’s involvement in water-related tasks, and the disruption to social cohesion at both the community and household level ultimately affect children’s health, learning, and ability to play. The dynamic and interrelated aspects of water insecurity and child health should be prioritized in future research, policy-making, and WASH interventions. Our findings can support the development of context-specific tools, such as a child water stress index for Palestine.

## Data Availability

The data from this study are unavailable due to the need to protect the privacy of the participants.

## Supplementary Materials

The following supporting information can be downloaded at: https://www.mdpi.com/article/10.3390/ijerph22020187/s1, (Consolidated Criteria for Reporting Qualitative Research) Checklist.

## Appendix A. Characteristics of FGD Participants and Location

	**Participants**	**Place of the FGD**	**Water Sources in the Community**
Pilot	5 women	Participant’s home	Piped water network, tanks
FGD1	11 women	Participant’s home	Tanks, spring
FGD2	3 women	Participant’s home	Tanks, well, rainwater
FGD3	6 women	Participant’s home	Tanks
FGD4	3 women	Council hall	Water network, tanks, protected well, spring
